# Post-ictal psychosis syndrome : A case report

**DOI:** 10.1192/j.eurpsy.2022.1146

**Published:** 2022-09-01

**Authors:** M. Moalla, N. Staali, E. Bergaoui, M. Zrelli, W. Melki

**Affiliations:** Razi Hospital, Psychiatry D, Manouba, Tunisia

**Keywords:** Psychosis, post-ictal, epilepsy

## Abstract

**Introduction:**

Psychiatric comorbidity is prevalent among patients with epilepsy. Post-ictal psychosis syndrome (PIP) is a recent entity important to know. It belongs to the group of epileptic psychoses. The clinical presentation is often atypical, and symptoms are usually related to seizures.

**Objectives:**

This work aimed to study the particularities of PIP.

**Methods:**

It is a case report of PIP, involving a patient hospitalized in psychiatry department.

**Results:**

We report the case of a 45-year-old woman, with medical history of generalized epilepsy which was stabilized under antiepileptic treatment (phenobarbital 150 mg/day). The patient was hospitalized for psychomotor instability and inconsistent speech after having experienced a generalized tonic-clonic seizure in the context of discontinuation of treatment. Psychiatric assessment revealed a hostility, a reluctance , a persecution delirium and auditory and visual hallucinations. A series of examinations have been carried out ; Neurological examination revealed no anomaly , a computed Tomography Scan of the Brain was normal. A lombar puncture was normal. A covid-19 infection was eliminated. The usual antiepileptic medication was reintroduced to the patient (Phenobarbital 150 mg/day),in association to benzodiazepines (clonazepam 4 mg/day). After 72 hours of treatment, psychiatric symptoms improved. The patient returned to its baseline condition after 7 days. A similar episode was reported two months earlier in the same circumstances with a similar symptomatology and a spontaneous resolution within 7 days.

**Conclusions:**

PPI syndrome, regardless of its good short-term prognosis, can potentially evolve into other psychiatric disorders of less good prognosis. Thus, this syndrome should be managed in collaboration with neurology and psychiatry.

**Disclosure:**

No significant relationships.

